# Childhood environmental harshness and unpredictability negatively predict eHealth literacy through fast life-history strategy

**DOI:** 10.3389/fpsyg.2023.1197189

**Published:** 2023-08-18

**Authors:** Wenfei Feng, Jinguang Zhang

**Affiliations:** ^1^School of Journalism and Communication, Sun Yat-sen University, Guangzhou, China; ^2^Center for Big Data and Public Communication, Sun Yat-sen University, Guangzhou, China

**Keywords:** eHealth literacy, eHEALS, environmental harshness, environmental unpredictability, life history theory

## Abstract

**Background:**

eHealth literacy—the ability to obtain, understand, evaluate, and use health information from the Internet—is important to maintaining and improving personal health. Prior research found that people differ notably in the levels of eHealth literacy, and this study tests a theoretical account of some of those individual differences. Drawing on life history theory, we propose that low eHealth literacy is partly the outcome of people adopting a resource-allocation strategy emphasizing early and fast reproduction, namely, a fast life-history strategy.

**Methods:**

We conducted a cross-sectional survey study (*N* = 1,036) that measured Chinese adult respondents’ eHealth literacy, childhood environmental harshness and unpredictability, and fast life-history strategy. Covariates included health-information seeking online, self-rated health, sex, age, education level, and monthly income.

**Results:**

Supporting a life-history explanation of eHealth literacy, childhood environmental harshness and unpredictability negatively predicted eHealth literacy through fast life-history strategy and mainly the insight-planning-control dimension of it. Harshness, not unpredictability, also directly and negatively predicted eHealth literacy after fast life-history strategy was controlled for.

**Conclusion:**

Our findings suggest that the psychological mechanisms associated with human life-history strategies produce at least some of the individual differences in levels of eHealth literacy, including those related to neuroticism, socioeconomic status, self-rated health and social capital. Thus, a possible way to increase future generation’s eHealth literacy and thereby their health is to reduce the harshness and unpredictability of the environment in which they grow up, thereby making them more likely to adopt a relatively slow life-history in their adulthood.

## 1. Introduction

eHealth literacy is “the ability to seek, find, understand, and appraise health information from electronic resources and apply the knowledge gained to addressing or solving a health problem” (Norman and Skinner, 2006a, p. 2). Based on this conceptualization, Norman and Skinner (2006b) developed an eight-item eHealth literacy scale named eHEALS. To date, eHEALS remains the most widely-used unidimensional, self-report measure of eHealth literacy, validated across 18 languages and 26 countries and regions (Lee et al., 2021).

With eHEALS, prior research found that levels of eHealth literacy positively correlated with the tendency to seek health information online (Manganello et al., 2016), levels of domain-specific health knowledge (Stellefson et al., 2018), quality of communication with doctors and levels of patient adherence (Lu and Zhang, 2021), and the likelihood of having a healthy lifestyle (Tsukahara et al., 2020; Xu et al., 2022). Given the importance of eHealth literacy to personal health, it would be of great theoretical and practical interest to explain the individual differences in levels of eHealth literacy identified in prior research (Norman and Skinner, 2006b; der Vaart et al., 2011; Neter and Brainin, 2012; Diviani et al., 2017; Del Giudice et al., 2018; Lwin et al., 2020; Magsamen-Conrad et al., 2020; Xu et al., 2020; Bergman et al., 2021; Cui et al., 2021; Wångdahl et al., 2021; Marsall et al., 2022; Tran et al., 2022). Doing so would help develop a general theory of eHealth literacy, identify individuals at risk of having low levels of eHealth literacy, and design interventions accordingly.

To this end, we tested in this study an evolutionary psychological account of eHealth literacy drawing on life history theory. Life history theory starts as an evolutionary biological theory (Stearns, 1992; Roff, 1993) but has been evoked by evolutionary psychologists to explain how a tradeoff between somatic versus reproductive effort underlies a wide range of individual differences in human personality traits and behavioral tendencies (Nettle and Frankenhuis, 2020). Drawing on the theory and relevant findings, we posit that low levels of eHealth literacy are partly the outcome of people adopting a fast life-history strategy. A fast life-history strategy prioritizes reproductive over somatic effort and is executed by high levels of present orientation, impulsivity, shifting and low levels of inhibition and self-control. While a fast life-history strategy and its underlying mechanisms are adaptive on average for people growing up in a harsh and/or unpredictable environment (Frankenhuis et al., 2016), they nevertheless weaken one’s motivation and ability to develop skillsets related to eHealth literacy. In what follows, we review past work on eHealth literacy, describe life history theory, elaborate a life-history explanation of eHealth literacy, and present data from a cross-sectional survey study conducted in China.

### 1.1. The concept of eHealth literacy

Norman and Skinner (2006a) argued in their lily model that eHealth literacy as the pistil develops from six petals of more basic types of literacy. These include traditional (i.e., basic reading and writing skills), information, media, health, scientific, and computer literacy. Among them, the first three are domain-general skillsets and constitute the analytic component of eHealth literacy. The remaining three are domain-specific capabilities and constitute the context-specific component of eHealth literacy. Both the analytic and the context-specific components are necessary for individuals to maximize the utility of information and communication technologies for personal health gains.

### 1.2. Correlates of eHealth literacy in prior work

Much prior research examined the demographic, personality, health, and social psychological correlates of eHealth literacy, and Table 1 summarizes recent findings.

**Table 1:** The demographic, personality, health, and social psychological correlates with eHealth literacy measured with eHEALS in recent studies.

Study	Sample characteristics	Method	Correlates
Norman and Skinner (2006b)	*N* = 664 Canadian adolescents	Zero-order correlations	Age (n.s.)
			Female (−)
			Self-rated health (n.s.)
			Technology use (n.s.)
Van der Vaart et al. (2013)	*N* = 496 Dutch adult patients	Zero-order correlations	Age (n.s.)
			Education (n.s.)
			Health-information seeking (+)
Neter and Brainin (2012)	*N* = 1,289 Israeli adults	Independent sample comparisons	Age (−)
			Education (+)
			Female (n.s.)
			Measured health (+)
			Information seeking (+)
			Self-rated health (n.s.)
Diviani et al. (2017)	*N* = 117 Italian-speaking Swiss adults	Zero-order correlations	Age (n.s.)
			Education (n.s.)
			Female (n.s.)
			Health-information seeking (+)
Del Giudice et al. (2018)	*N* = 868 Italian adults	Zero-order correlations	Age (+)
			Education (+)
			Health-information seeking (+)
			Self-rated health (+)
Tennant et al. (2015)	*N* = 283 U.S. adults over 50	Multiple regression	Age (−)
			Education (+)
			Health-information seeking (+)
Lwin et al. (2020)	*N* = 923 Chinese adults	Zero-order correlations	Age (+)
			Education (+)
			Female (n.s.)
			Health-information seeking (+)
			Income (+)
Magsamen-Conrad et al. (2020)	*N* = 525 U.S. adults	Zero-level correlations and multiple regression	Education (+)
			Health-information seeking (+)
Xu et al. (2020)	*N* = 574 Chinese adult patients	Zero-order correlations	Age (−)
			Being employed (+)
			Education (+)
Cui et al. (2021)	*N* = 1,021 Chinese elderlies	Zero-order correlations and multiple regression	Structural social capital (+)
Wångdahl et al. (2021)	*N* = 298 adult Arabic Swedes	Zero-order correlations	Age (n.s.)
			Education (+)
			Internet use (+)
			Self-rated health (+)
Bergman et al. (2021)	*N* = 703 adult Swedes	Multiple logistic regression	Age (−)
			Education (+)
			Female (+)
			Internet use (+)
			Self-rated health (+)
Marsall et al. (2022)	*N* = 470 adult Germans	Zero-order correlations	Age (+)
			Education (n.s.)
			Female (n.s.)
			Impulsivity (n.s.)
			Neuroticism (−)
			Self-rated health (+)
Tran et al. (2022)	*N* = 1,851 Vietnamese nursing students	Multiple regression	Age (n.s.)
			Female (+)
			Socioeconomic status (+)

The plus sign (+) represents “positive correlation”, the minus sign (−) represents “negative correlation,” and n.s. represents non-significant.

From Table 1, socioeconomic status (e.g., education attainment, income, and employment status) (10 out of 13 effects), self-rated health (4 out of 6 effects), and Internet use (9 out of 10 effects) appear robust correlates of eHealth literacy. In comparison, the effects of age and sex were more mixed, with at least half of them being non-significant. Also noticeable are neuroticism negatively whereas social capital (e.g., social connections and support) positively correlated with eHealth literacy.

These findings provided much insight into the sources of the individual difference in the levels of eHealth literacy. However, an even better understanding of eHealth literacy may be gained by explaining *why*, for example, socioeconomic status, self-rated health, and social capital positively whereas neuroticism negatively correlates with eHealth literacy. This explanation entails identifying the psychological mechanisms that underlie the motivation and ability to acquire eHealth literacy, and this research aims to do so by drawing on life history theory.

### 1.3. Life history theory

Life history theory (at least the evolutionary psychological version of it) concerns how organisms allocate resources between three basic life activities, namely physical maintenance, growth, and reproduction (Nettle and Frankenhuis, 2020). Physical maintenance refers to building and repairing the digestive, immune, and other systems central to survival, growth refers to physical and cognitive development (i.e., embodied capital) (Kaplan et al., 2009), and reproduction refers to activities directly related to passing on one’s own copies of genes (e.g., mate competition and courtship). Physical maintenance and growth are collectively called somatic effort (Griskevicius et al., 2011).

#### 1.3.1. Slow and fast life-history strategies

Individuals differ in when to prioritize somatic effort versus reproduction, and different prioritization decisions entail trade-offs (Bolund, 2020). At one extreme, people may heavily invest in somatic effort at the expense of reproduction. Doing so helps increase one’s physical and socioeconomic competence for better future reproductive opportunities and offspring quality but tends to decrease offspring quantity and incur the risk of death before first reproduction.

At the other extreme, one may trade somatic effort for reproduction. Doing so helps maximize offspring quantity and reduce the risk of death before first reproduction. However, reproducing early and fast diverts resources away from maintenance and/or growth, decreasing one’s own physical and socioeconomic competence and the quality of their offspring.

These two allocation strategies—prioritizing somatic effort or reproduction—are, respectively, called a slow or a fast life-history strategy (Del Giudice, 2020). Because somatic effort prepares one for future reproduction, a slow life-history strategy is also said to focus on future reproduction, whereas a fast life-history strategy is said to focus on current reproduction. These two “pure” strategies bound a continuum with mixed strategies falling in between.

#### 1.3.2. Determinants and outcomes of adopting slower or faster life-history strategies

The harshness and unpredictability of one’s early developmental (i.e., childhood) environment are two important determinants of people adopting a slower or faster life-history strategy (Ellis et al., 2022). Environmental harshness describes the average level of extrinsic threats (e.g., violence and infectious diseases) present in one’s environment, whereas environmental unpredictability indexes the degree of temporal and spatial variation of that average (Brumbach et al., 2009; Young et al., 2020). Higher childhood environmental harshness and/or unpredictability tend to cause higher levels of—and variation in—adult mortality. A fast life-history strategy helps mitigate those mortality threats by motivating people to reproduce early, many and in short intervals (Frankenhuis et al., 2016; Ellis et al., 2022).

Prior research has revealed a suite of psychological mechanisms that help execute a fast life-history strategy due to people growing up in a harsh and/or unpredictable environment. For example, both childhood environmental harshness and unpredictability have been found to negatively predict school performance and positively predict present orientation, impulsivity, and risk proneness either directly or through fast life-history strategy (Maner et al., 2017; Chang and Lu, 2018; Chang et al., 2019; Maranges and Strickhouser, 2022).

Prior research has also demonstrated the unique effects of childhood environmental harshness and unpredictability on outcome variables of interest. For example, harshness was found to negatively predict sense of control and positively predict present orientation and risk diversification under primes of mortality or financial threats (Griskevicius et al., 2011; White et al., 2013; Mittal and Griskevicius, 2014). Maranges and Strickhouser (2022) found that harshness, not unpredictability, positively predicted the earliness and number of romantic relationships one would have.

On the other hand, unpredictability—not harshness—was found to negatively predict physical health in adulthood, altruistic concerns, and the tendency to adopt a deliberate cognitive style but positively predict the number of sex partners, aggressiveness, and the desire for instant gratification (Brumbach et al., 2009; Simpson et al., 2012; Maranges and Strickhouser, 2022; Martinez et al., 2022; Wang et al., 2022). Notably, Mittal et al. (2015) found that unpredictability but not harshness negatively predicted inhibition (i.e., the ability to override dominant responses) but positively predicted shifting (e.g., the ability to switch among different tasks), especially under primes of economic uncertainty.

### 1.4. A life-history explanation of eHealth literacy

Because fast life-history strategists—as a result of growing up in a harsher and/or unpredictable environment—would tend to trade somatic effort (e.g., physical maintenance) for reproduction, they should generally be less motivated to engage in health-promoting behaviors (Tybur et al., 2012; Cabeza de Baca et al., 2016). Supporting this hypothesis, childhood environmental harshness was found to negatively predict health effort (Mell et al., 2018) and the desire for health insurance under threats of financial instability (Mittal and Griskevicius, 2016). Also, childhood social disadvantage (e.g., relatively low socioeconomic status and unstable family life) was found to positively predict engaging in health risk behaviors including smoking and excessive drinking (Non et al., 2016).

eHealth literacy comprises skillsets conducive to maintaining and improving personal health (see Section 1), and developing eHealth literacy is thus by definition a health-promoting behavior. Therefore, life history theory predicts that *fast (relative to slow) life-history strategists would generally be less motivated to develop—and thus have lower levels of—eHealth literacy*. Regarding mechanism, the multiple skillsets necessary to developing eHealth literacy (see Section 1.1) are unlikely to acquire overnight but entail sustained and disciplined investment of cognitive resources into relevant areas of learning and practicing. Specifically, developing eHealth literacy likely includes (1) gaining knowledge on health in general and/or particular diseases (Green, 2022; Lee et al., 2022), (2) distinguishing between sources of different levels of credibility (Chang et al., 2021), (3) evaluating the usefulness of information (Kim et al., 2021), (4) comparing the potential outcomes of alternative pieces of advice (Li et al., 2021), and (5) executing a chosen course of action with persistence (Xu et al., 2022). Those activities invariably demand planning, patience, and self-control for one to accrue benefits (e.g., physical health) over time. Indeed, Norman and Skinner (2006b) argued that the concept of eHealth literacy partly builds on the notion of self-efficacy, which is the belief in one’s own ability to carry out a desired course of action and at the center of human agency that entails planning, forethought, self-motivation, and self-monitoring (Bandura, 2004).

On average, fast (relative to slow) life-history strategists lack planning, patience, and self-control. Planning is a core dimension of slow life-history strategy (Figueredo et al., 2006). At the same time, lower levels of inhibition and higher levels of shifting, impulsivity, an intuitive cognitive style, and present orientation—all of which are manifestations of a faster life-history strategy (see Section 1.3.2)—would make one prone to distraction, act based on intuition rather than on deliberation, focus on immediate rewards, and lack self-control. While those traits would help execute a fast life-history strategy, thereby increasing one’s reproductive success in a harsh and/or unpredictable environment, they would nevertheless be counter-effective to cultivating eHealth literacy. These considerations led us to predict that childhood environmental harshness (Prediction 1a) and unpredictability (Prediction 1b) would negatively predict eHealth literacy through fast life-history strategy.

Harshness and unpredictability may also directly and negatively predict eHealth literacy. For example, growing up in a harsh environment tends to deprive one of their accesses to media and computers and even education. This would thwart the development of several if not all of the basic types of literacy that eHealth literacy entails. Meantime, the stochastic changes of an environment would disrupt information gathering (e.g., sources of information become unavailable), comprehension (e.g., emerging challenges distract one from studying gathered information), evaluation (e.g., novel environmental features render comprehended information useless), and use (e.g., unforeseen circumstances force one to change plans). These considerations lead us to predict that childhood environmental harshness (Prediction 2a) and unpredictability (Prediction 2b) would negatively and directly predict eHealth literacy after controlling for fast life-history strategy.

## 2. Method

### 2.1. The sample

We recruited 1,082 Chinese adult Internet users to participate in the study for a small payment. The study was conducted in Chinese in November, 2021 via *Jishu Yun*,^1^ a China-based company that facilitates online academic research. We dropped 46 respondents who reported to be under 18 years for ethical considerations, leaving the final *N* = 1,036. The final sample consisted of 59.7% female, had an average age of 34.8 years (SD = 8.10; range = 18–65), had a median education level of completing senior high school, and had a median income level of earning between 4,500 and 6,000 RMB per month.

### 2.2. Procedure and measures

After providing informed consent, respondents were asked to complete the eight-item eHEALS scale on eHealth literacy (1 *strongly disagree*, 7 *strongly agree*) (Norman and Skinner, 2006b). The scale showed satisfactory internal consistency (Cronbach’s *α* = 0.88) with larger scores indicating higher levels of eHealth literacy. Respondents were then asked to complete a four-item measure of seeking health information online (e.g., “I have sought out health information on the Internet”; 1 *strongly disagree*, 7 *strongly agree*) (Lagoe and Atkin, 2015). The scale showed satisfactory internal consistency (Cronbach’s *α* = 0.76) with larger values indicating more frequent health-information seeking online. We included this variable to control for respondents’ experience with using the Internet for health purposes.

Next, respondents were asked to complete the Mini-*K* scale that comprises 20 cognitive and behavioral indicators of a slow life-history strategy (Figueredo et al., 2006). The scale contains seven dimensions (Figueredo et al., 2014), namely (1) insight, planning, and control (e.g., “I can often tell how things will turn out”), (2) mother and father relationship quality (e.g., “When growing up, I had a close and warm relationship with my biological mother”), (3) family contact and support (e.g., “I am often in social contact with my blood relatives”), (4) friends contact and support (e.g., “I am often in social contact with my friends”), (5) secure romantic partner attachment (e.g., “I have a close and warm romantic relationship with my sexual partner”), (6) general social altruism (e.g., “I often give emotional support and practical help to my friends”), and (7) religiosity (e.g., “I am closely connected to and involved in my religion”) (1 *strongly disagree*, 7 *strongly agree*). Mini-*K* has been widely used in prior work and demonstrated sufficient validity assessed with diverse samples (Dunkel and Decker, 2010; Figueredo et al., 2015; Richardson et al., 2017; Kiire, 2020) but for a recent critique of the approach underlying the development and use of scales such as the Mini-*K* (Richardson et al., 2017; Međedović, 2020; Sear, 2020; Richardson et al., 2021).

From the scale, we dropped the three questions on secure romantic partner attachment because they had low inter-item reliability in our prior work conducted in China. We also dropped the question on religiosity because it likely has low variance in China, and did not include the item on relationship with father due to a programming oversight. The remaining 15 items showed satisfactory internal consistency (Cronbach’s *α* = 0.86). We computed the averages of the items, and reversed the averages by subtracting them from 8 so that larger values indicate a *faster* life-history strategy.

From the 15 items of Mini-*K*, we further computed the average scores of the six items^2^ pertaining to insight, planning, and control (henceforth IPC for short; Cronbach’s *α* = 0.71). This is because our hypothesis suggests that childhood environmental harshness and unpredictability would negatively predict levels of eHealth literacy through mechanisms related to present orientation, impulsivity, and lack of self-control (Section 1.4). We computed the average scores of the remaining items to index non-IPC (Cronbach’s *α* = 0.80). This is because, at least with our sample, the reliability of the other dimensions of Mini-*K* (e.g., parent relationship quality, family contact and support, friends contact and support, and general altruism) was low (Cronbach’s αs < 0.60). We reversed both IPC and non-IPC to make them consistent with our measure of fast life-history strategy.

After completing mini-*K*, respondents were asked to complete a nine-item scale of childhood environmental unpredictability (e.g., “my family life was generally inconsistent and unpredictable from data-to-day”; 1 *strongly disagree*, 7 *strongly agree*) (Mittal et al., 2015). The scale showed satisfactory internal consistency (Cronbach’s = 0.89) with larger values indicating higher levels of unpredictability. Next, respondents were asked to complete a four-item scale of childhood environmental harshness [e.g., “my family usually had enough money for things when I was growing up” (reversed); 1 *strongly disagree*, 7 *strongly agree*] (Griskevicius et al., 2011). The scale showed satisfactory internal consistency (Cronbach’s = 0.84). We reverse-coded the scale so that larger values indicated higher levels of harshness.

Lastly, respondents were then asked to rate their current health (1 *very unhealthy*, 5 *very healthy*), and provide information on their age, sex, education level, and monthly income.

## 3. Results

### 3.1. Descriptive statistics, data preparation, and analytic strategies

We presented descriptive statistics and inter-correlations in Table 2.

**Table 2:** Descriptive statistics and intercorrelations of main variables.

Variable	*M*	SD	1	2	3	4	5	6	7	8
1. eHealth	4.77	1.08								
2. Fast LH	2.64	0.80	−0.56**							
3. Harshness	3.84	1.33	−0.35**	0.28**						
4. Unpredictability	3.66	1.24	−0.11**	0.21**	−0.09**					
5. Seeking	4.99	1.09	0.67**	−0.60**	−0.34**	−0.12**				
6. Health	4.10	0.82	0.28**	−0.29**	−0.28**	−0.09**	0.16**			
7. Age	34.81	8.10	−0.13**	−0.04	0.10**	0.02	−0.04	−0.08**		
8. Education	2.47	1.10	0.11**	−0.14**	−0.09**	−0.15**	0.11**	0.12**	−0.40**	
9. Income	3.79	1.22	0.07*	−0.24**	−0.03	−0.21**	0.15**	0.10**	−0.08*	0.48**

*M* and SD are mean and standard deviation, respectively. eHealth, eHealth literacy; Fast LH, fast life-history strategy; harshness, childhood environmental harshness; unpredictability, childhood environmental unpredictability; seeking, health-information seeking online; and health, self-rated health. *p < 0.05, **p < 0.01.

For hypothesis testing, we performed path analyses in Mplus Version 8 (Muthén et al., 2017), and controlled for respondents’ age, sex and levels of education and income, self-rated health, and health-information seeking online given prior findings summarized in Table 1. We Z-transformed all variables so that raw regression coefficients indexed effect sizes.

### 3.2. Hypothesis testing

**Figure 1:**
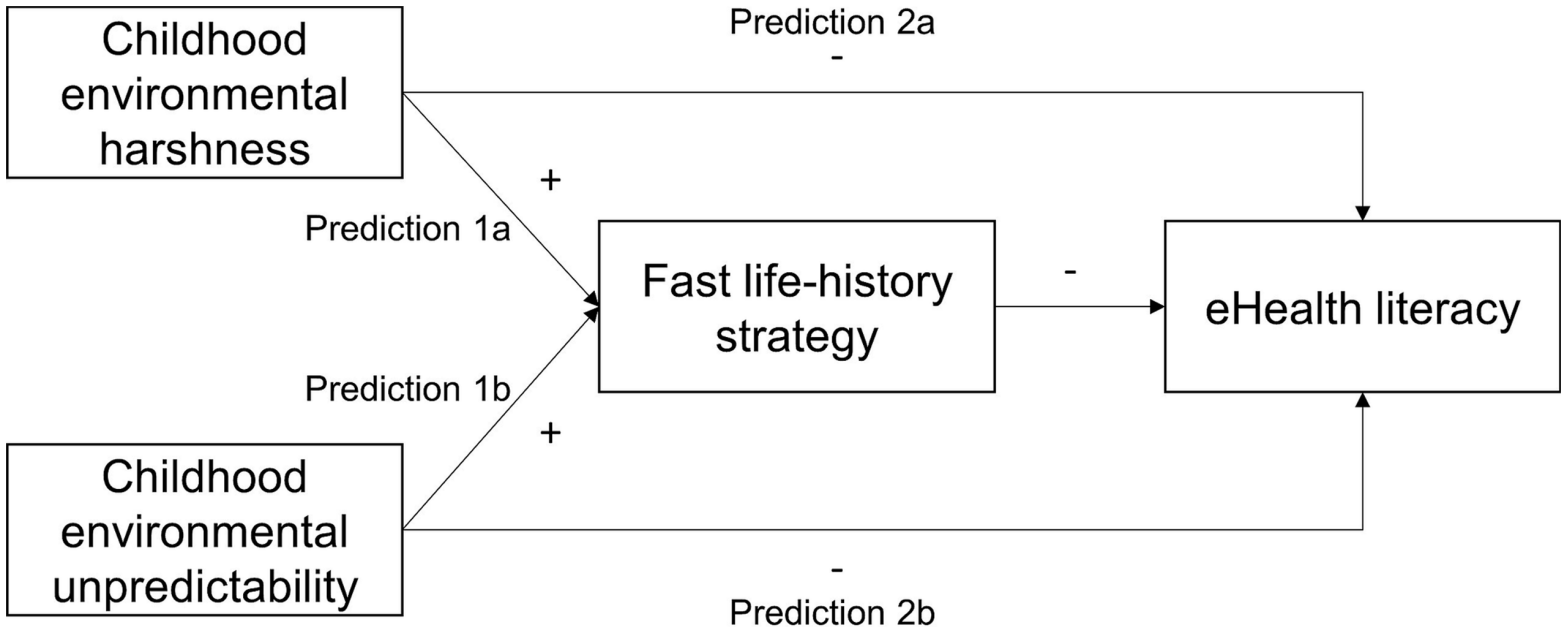
The schematic representation of prediction sets 1 and 2. The plus sign (+) reads “positively predicts” and the negative sign (−) reads “negatively predicts”.

**Figure 2:**
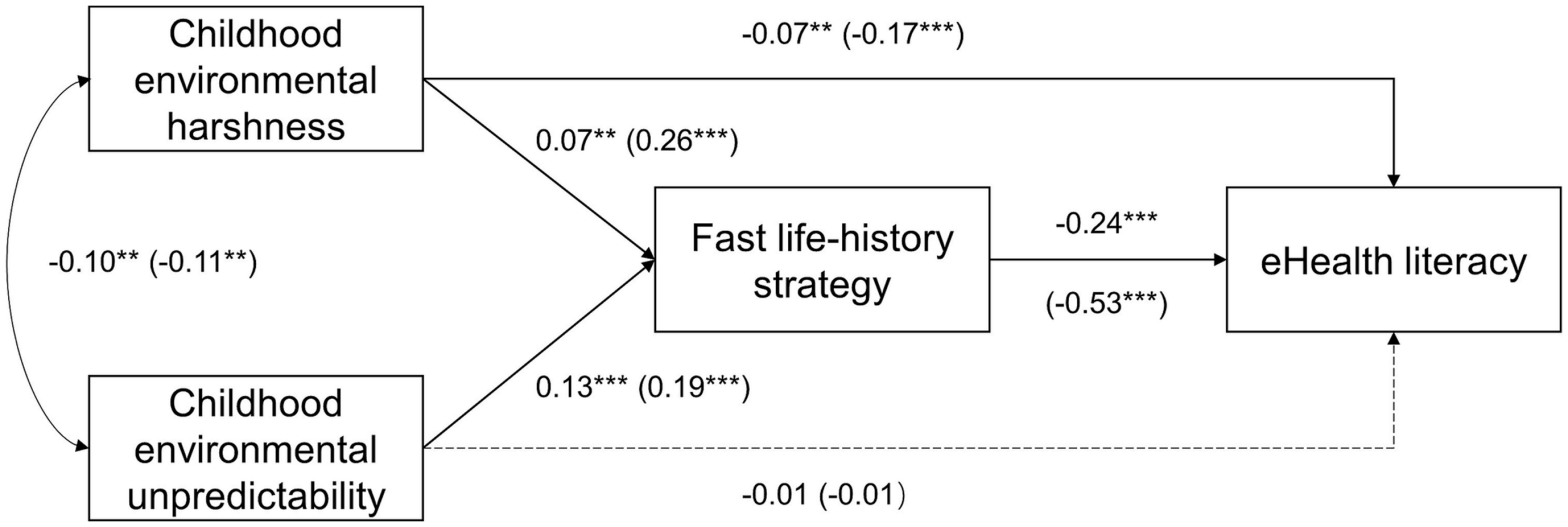
Main findings of path models testing prediction sets 1 and 2. Dash lines represent non-significant paths. Parameters in parentheses were from the model that dropped health-information seeking online as a covariate. **p < 0.01; ***p < 0.001.

We predicted that childhood environmental harshness and unpredictability would negatively predict eHealth literacy through fast life-history strategy (Predictions 1a and 1b) and directly (Predictions 2a and 2b). We estimated a path model as depicted in Figure 1 to test those predictions while controlling for the covariates mentioned above. The main findings were presented in Figure 2, and detail results were summarized in Supplementary Table S1.

As predicted, harshness and unpredictability positively and significantly predicted fast life-history strategy, which negatively and significantly predicted eHealth literacy. The indirect effect of harshness was ß = −0.02, with the 95% bias-corrected confidence interval (CI) based on 10,000 resamples estimated (−0.03, −0.01). The indirect effect of unpredictability was ß = −0.03, with the 95% CI estimated (−0.04, −0.02). These results supported Predictions 1a and 1b.

Also as predicted, harshness—after we controlled for fast life-history strategy, unpredictability, and all covariates—negatively and significantly predicted eHealth literacy. However, the direct correlation between unpredictability and eHealth literacy was not significant. These results supported Prediction 2a but not Prediction 2b.

Further, health-information seeking online and self-rated of health positively—whereas age and income negatively—predicting eHealth literacy. There was no evidence that respondents’ sex or education level predicted eHealth literacy.

### 3.3. Robustness and exploratory tests

eHealth literacy, health-information seeking online, and fast life-history strategy showed large correlations (|*r*| > 0.56) (Table 2), and we thus checked whether there was collinearity when predicting eHealth literacy from health-information seeking online and fast life-history strategy in an OLS regression model. There was no evidence for collinearity, as the VIFs were estimated 1.63 for both predictor variables and well below the conventional threshold value (i.e., 10). Next, we re-ran the analysis in Section 3.2 while dropping health-information seeking online as a covariate. As Figure 2 showed, most path coefficients increased in size but the statistical conclusions remained the same. The indirect effect of harshness was now estimated ß = −0.14, with the 95% CI estimated (−0.18, −0.11), and that of unpredictability was estimated ß = −0.10, with the 95% CI estimated (−0.13, −0.07). Details of the model were in Supplementary Table S2. We then explored whether the indirect effects of harshness and unpredictability on eHealth literacy was mainly through IPC (i.e., insight, planning, and control). To do so, we estimated the following path model while controlling for all covariates specified in Section 3.1.

**Figure 3:**
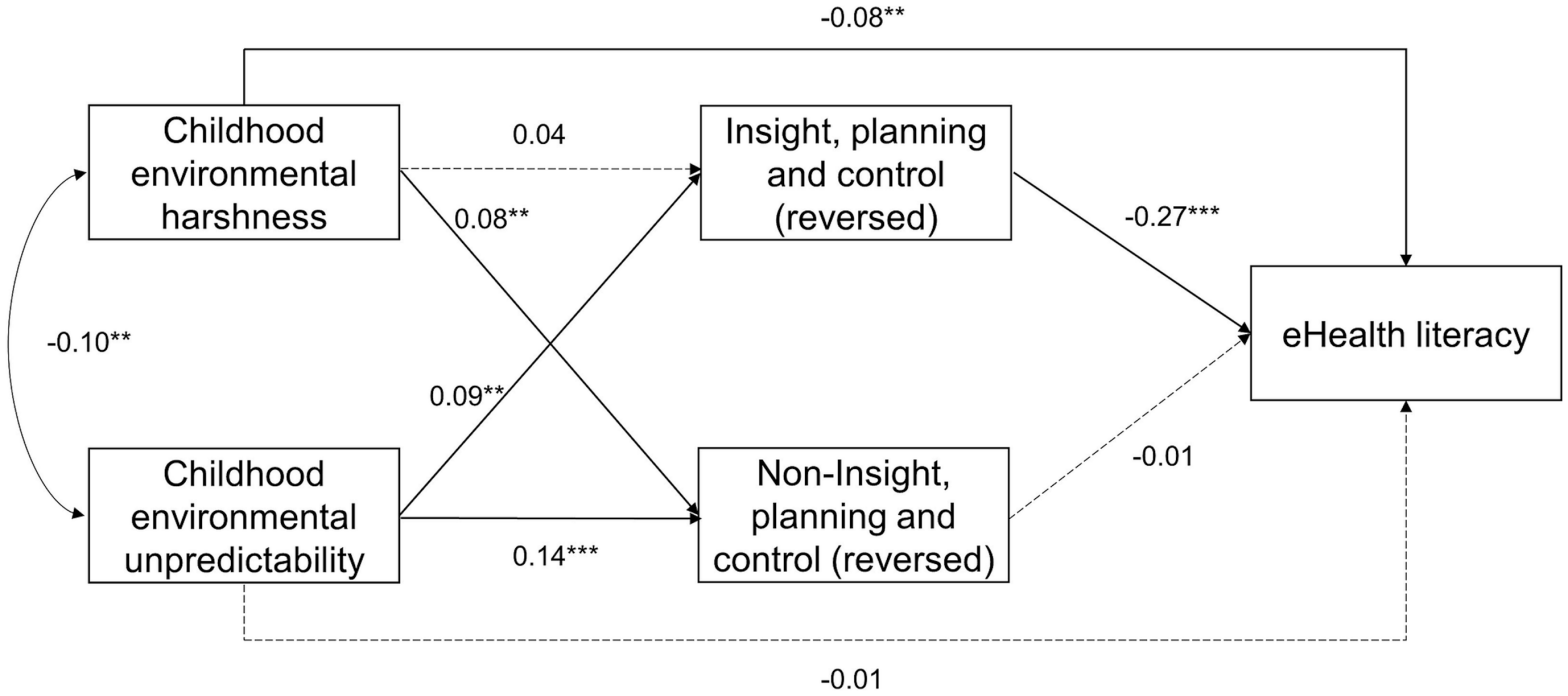
Main findings of a path model exploring the mediating effects of IPC and non-IPC. IPC, insight, planning, and control. Both IPC and non-IPC were reversed to indicate a fast life-history strategy. Dash lines represent non-significant paths. **p < 0.01; ***p < 0.001.

As Figure 3 showed, IPC (reversed) but not non-IPC (reversed) negatively and significantly predicted eHealth literacy. Similar to using fast life-history strategy as the mediator (Figure 2), harshness but not unpredictability directly and negatively predicted eHealth literacy after we controlled for IPC (reversed) and non-IPC (reversed). Unlike the model in Figure 2, however, the indirect effect of harshness through IPC (reversed) was not significant, ß = −0.01, with the 95% CI estimated (−0.03, 0.002). The indirect effect of unpredictability through IPC (reversed) remained significant, ß = −0.02, with the 95% CI estimated (−0.04, −0.01). Details of the model were in Supplementary Table S3. These results further supported our life-history hypothesis of eHealth literacy by clarifying the underlying mechanisms.

## 4. Discussion

In this study, we tested a life-history explanation of eHealth literacy in a cross-sectional survey study with a reasonably large sample of Chinese adults. As predicted, childhood environmental harshness and unpredictability negatively predicted eHealth literacy through fast life-history strategy after controlling for relevant covariates. When we divided the measure of fast life-history strategy into IPC (i.e., insight, planning, and control) and non-IPC, IPC but not non-IPC predicted eHealth literacy and mediated the correlation between childhood environmental unpredictability (but not harshness) and eHealth literacy. In contrast, after controlling for fast life-history strategy (or IPC reversed) and covariates, harshness but not unpredictability directly and negatively predicted eHealth literacy. These findings largely supported the life-history explanation of eHealth literacy.

### 4.1. Implications for demographic and health correlates of eHealth literacy

Prior research found that socioeconomic status (e.g., education attainment, income, and employment status) and self-rated health positively predicted eHealth literacy (Table 1). A likely reason is that both higher socioeconomic status and better self-rated health are correlates of a slower life-history strategy (Brumbach et al., 2009; Mell et al., 2018). Indeed, we found that education, income, and self-rated health all negatively correlated with *fast* life-history strategy in zero-order correlations (Table 2) and—with the exception of education—in the path model (Supplementary Tables S1–S3).

As in prior studies, we found that education, income, and self-rated health all positively correlated with eHealth literacy in zero-order correlations (Table 2). However, after we controlled for fast life-history strategy and its two environment determinants in the path models, education no longer predicted eHealth literacy, and income emerged as a negative predictor (Supplementary Tables S1–S3). These results suggest that life-history variables accounted for the associations between education and income on the one hand and eHealth literacy on the other. In comparison, self-rated health in the path model remained a positive predictor of eHealth literacy. Thus, other mechanisms than life-history strategies may also cause the association between self-rated health and eHealth literacy, and future research may consider identifying those mechanisms.

### 4.2. Implications for personality correlates of eHealth literacy

Prior research found that neuroticism negatively correlated with eHealth literacy (Marsall et al., 2022). We did not measure neuroticism in this study, but neuroticism—especially anxiety—has been found to positively correlate with fast life-history strategy (Davis et al., 2019). This may partly be why neuroticism negatively correlated with eHealth literacy.

Notably, Marsall et al. (2022) found no evidence that impulsivity correlated with eHealth literacy. This finding appears inconsistent with our life-history explanation, which posits that fast (relative to slow) life-history strategists tend to have lower eHealth literacy partly due to being impulsive. As one possibility, impulsivity *does* negatively correlate with eHealth literacy but Marsall et al. (2022) somehow did not detect it (e.g., a Type-II error). Another possibility is that the mechanism linking fast life-history strategy and low eHealth literacy is less about one being impulsive than about one diverting cognitive and material resources away from health-promoting behaviors. Thus, future research may consider carefully testing the mechanisms postulated by our life-history account of eHealth literacy.

### 4.3. Implications for social psychological correlates of eHealth literacy

Prior research found that structural social capital and Internet use (for health purposes) positively correlated with eHealth literacy (Table 1). Because stronger social connectedness and support are dimensions of a slow life-history strategy (Figueredo et al., 2006), Cui et al. (2021)’s finding is consistent with our life-history explanation of eHealth literacy. To the extent that eHealth literacy concerns the ability to obtain health information from the Internet, that eHealth literacy positively correlates with health-information seeking online is perhaps not surprising. However, our finding that fast life-history strategy negatively correlated with health-information seeking online (*r* = −0.60; Table 2) suggests an intriguing possibility. That is, similar to higher levels of eHealth literacy, the tendency to seek health information from the Internet may also be a form of somatic investment and thus an outcome of people adopting a slow life-history strategy. In fact, that both eHealth literacy and health-information seeking online negatively correlated with mini-*K* with large effect sizes (|*r*| > 0.50; Table 2) suggests that those two variables reflect a common underlying construct, which could be health effort (Mell et al., 2018). Future research may consider formally testing this possibility, thereby adding to the growing literature on life-history theory and health psychology (Tybur et al., 2012).

### 4.4. Limitations and future research

First, we used eHEALS to measure eHealth literacy, but eHEALS has been subject to debates over its content validity (e.g., missing Web 2.0 and online multimedia) (Van der Vaart et al., 2013; Paige et al., 2018). Over the years, new scales have been developed to measure eHealth literacy (Lee et al., 2021), which poses the question of to what extent the results of this study would hold when using those newer scales of eHealth literacy. Relatedly, there have also been debates on the validity of what has been known as the psychometric approach to studying human life history strategies (Richardson et al., 2017; Međedović, 2020; Sear, 2020; Richardson et al., 2021). Thus, future research may consider replicating our study with biodemographic (e.g., age of first sexual intercourse, number of offspring) (Sear, 2020) in addition to psychometric indicators of life history strategies.

Second, the measures of childhood environmental harshness and unpredictability were retrospective in nature and might have subject to the influence of personal memory. Thus, future research may consider using a panel design that uses objective measures of one’s childhood environment and measures respondents’ eHealth literacy at later stage of the study. This design would also help establish the causal effects—if any—of environmental features on levels of eHealth literacy.

At the same time, the measure of childhood environmental harshness varied considerably from study to study. In the studies cited in this research, for example, that variable was indexed by extrinsic mortality risks (Brumbach et al., 2009; Chang et al., 2019), resources availability (Griskevicius et al., 2011; White et al., 2013; Mittal and Griskevicius, 2014; Maner et al., 2017; Chang and Lu, 2018; Martinez et al., 2022), and parent education attainment, income, employment status, and neighborhood socioeconomic status (Simpson et al., 2012; Maranges and Strickhouser, 2022). In comparison, at least in the studies cited above, childhood environmental unpredictability was mostly indexed by chaotic home life (e.g., the unpredictable absence of a parent) (Brumbach et al., 2009; Mittal et al., 2015; Maner et al., 2017; Chang and Lu, 2018; Chang et al., 2019; Wang et al., 2022). Notably, though, other measures of unpredictability included changes in parent’s employment status, household composition, frequency of moving, and inconsistencies in life outside home (e.g., school, neighborhood) (Simpson et al., 2012; Maranges and Strickhouser, 2022; Martinez et al., 2022).

We measured harshness and unpredictability, respectively, as resource availability and chaotic home life because they appeared the most common choices in the work we reviewed for this study. Importantly, the studies that adopted those measures concern the likely mechanisms (e.g., present orientation, an intuitive thinking style, inhibition and shifting, and sense of control) underlying the association between life-history strategies and levels of eHealth literacy. In other words, prior findings formed the basis of our operational decisions. However, future research might consider using updated measures of harshness and unpredictability (Maranges et al., 2022) to replicate and extend this study. This would offer more valid tests of our life-history hypothesis of eHealth literacy.

Lastly, future research may consider testing the life-history explanation of eHealth literacy with respondents from other countries and regions of the world than China. We are confident in the validity of our hypothesis because both life history theory and eHEALS have been validated cross-culturally. However, the cross-cultural validity of the life-history explanation of eHealth literacy remains to be verified with empirical data.

## 5. Conclusion

This study found that Chinese people who grew up in a harsher and/or more unpredictable environment were more likely to have lower levels of eHealth literacy and this was partly because they had lower levels of insight, planning, and self-control. Those findings suggest that the psychological mechanisms associated with human life-history strategies produce at least some of the individual differences in the levels of eHealth literacy. Thus, a possible way to increase future generations’ eHealth literacy is to reduce the harshness and unpredictability of their childhood environments, thereby increasing the likelihood of them adopting a slow life-history strategy.

## Data availability statement

The original contributions presented in the study are publicly available. This data can be found here: https://osf.io/yct4w/?view_only=85ba790d30644db58b2875a7be99868c.

## Ethics statement

Ethical approval was not required for the studies involving humans because the institution the authors are affiliated with only requires ethical approval for biomedical research. The studies were conducted in accordance with the local legislation and institutional requirements. The participants provided their written informed consent to participate in this study.

## Author contributions

WF conceived and designed the study and collected data. WF and JZ analyzed data and wrote the manuscript and reviewed the manuscript. All authors contributed to the article and approved the submitted version.

## Funding

This research was supported by Sun Yat-sen University’s startup fund to JZ, (grant no: 17000-12230014).

## Conflict of interest

The authors declare that the research was conducted in the absence of any commercial or financial relationships that could be construed as a potential conflict of interest.

## Publisher’s note

All claims expressed in this article are solely those of the authors and do not necessarily represent those of their affiliated organizations, or those of the publisher, the editors and the reviewers. Any product that may be evaluated in this article, or claim that may be made by its manufacturer, is not guaranteed or endorsed by the publisher.

## Footnotes

^1^www.databnu.com

^2^They are the first six items on the standard Mini-*K* scale: (1) “I can often tell how things will turn out”; (2) “I try to understand how I got into a situation to figure out how to handle it”; (3) “I often find the bright side to a bad situation”; (4) “I do not give up until I solve my problems”; (5) “I often make plans in advance”; and (6) “I avoid taking risks”.
