# Supplementary material for: Childhood environmental harshness and unpredictability negatively predict eHealth literacy through fast life-history strategy
Source: Front Psychol. 2023 Aug 18;14:1197189. doi: 10.3389/fpsyg.2023.1197189 (PMC10473102; doi:10.3389/fpsyg.2023.1197189)
Supplement: Supplementary file 1 [file Data_Sheet_1.docx]

Supplemental Information

To

Childhood Environmental Harshness and Unpredictability Negatively Predict eHealth Literacy through Fast Life-History Strategy

**Table S1**

*Results of path model controlling for health-information seeking online with bootstrapped standard errors with 10,000 resamples*

| Parameters | ß | *SE* | *Z* | *p* |
| --- | --- | --- | --- | --- |
| *Predicting eHealth literacy* (outcome) |  |  |  |  |
| **Fast life-history strategy** | -0.24 | 0.03 | -7.59 | < .001 |
| **Harshness** | -0.07 | 0.03 | -2.70 | .007 |
| **Unpredictability** | -0.01 | 0.02 | -0.34 | .74 |
| Health-information seeking online | 0.51 | 0.04 | 13.76 | < .001 |
| Self-rated health | 0.10 | 0.03 | 3.97 | < .001 |
| Sex (1 = male, 2 = female) | -0.04 | 0.02 | -1.78 | .08 |
| Age | -0.10 | 0.03 | -3.96 | < .001 |
| Education | -0.01 | 0.03 | -0.46 | .65 |
| Income | -0.09 | 0.03 | -3.20 | .001 |
| *Predicting fast LH strategy* (mediator) |  |  |  |  |
| **Harshness** | 0.07 | 0.03 | 2.95 | .003 |
| **Unpredictability** | 0.13 | 0.02 | 5.43 | < .001 |
| Health-information seeking online | -0.54 | 0.03 | -15.72 | < .001 |
| Self-rated health | -0.18 | 0.03 | -6.19 | < .001 |
| Sex (1 = male, 2 = female) | -0.00 | 0.02 | -0.19 | .85 |
| Age | -0.10 | 0.03 | -3.22 | .001 |
| Education | -0.02 | 0.03 | -0.60 | .55 |
| Income | -0.11 | 0.03 | -4.30 | < .001 |

*Note*: Key predictor variables are in bold.

**Table S2**

*Results of path model without controlling for health-information seeking online with bootstrapped standard errors with 10,000 resamples*

| Parameters | ß | *SE* | *Z* | *p* |
| --- | --- | --- | --- | --- |
| *Predicting eHealth literacy* (outcome) |  |  |  |  |
| **Fast life-history strategy** | -0.53 | 0.03 | -19.22 | < .001 |
| **Harshness** | -0.17 | 0.03 | -5.07 | < .001 |
| **Unpredictability** | -0.01 | 0.03 | -0.46 | .65 |
| Self-rated health | 0.07 | 0.03 | 2.34 | .02 |
| Sex (1 = male, 2 = female) | -0.02 | 0.03 | -0.84 | .40 |
| Age | -0.12 | 0.03 | -4.00 | < .001 |
| Education | -0.02 | 0.03 | -0.50 | .62 |
| Income | -0.08 | 0.03 | -2.64 | .008 |
| *Predicting fast LH strategy* (mediator) |  |  |  |  |
| **Harshness** | 0.26 | 0.03 | 8.64 | < .001 |
| **Unpredictability** | 0.19 | 0.03 | 6.90 | < .001 |
| Self-rated health | -0.20 | 0.03 | -6.13 | < .001 |
| Sex (1 = male, 2 = female) | -0.03 | 0.03 | -0.16 | .25 |
| Age | -0.10 | 0.04 | -2.95 | .003 |
| Education | -0.02 | 0.04 | -0.62 | .53 |
| Income | -0.18 | 0.03 | -5.47 | < .001 |

*Note*: Key predictor variables are in bold.

**Table S3**

*Results of path model predicting eHealth literacy from IPC (reversed) and non-IPC (reversed) with bootstrapped standard errors with 10,000 resamples*

| Parameters | ß | *SE* | *Z* | *p* |
| --- | --- | --- | --- | --- |
| *Predicting eHealth literacy* (outcome) |  |  |  |  |
| **IPC (reversed)** | -0.27 | 0.04 | -7.46 | < .001 |
| **non-IPC (reversed)** | -0.11 | 0.03 | -0.35 | .72 |
| **Harshness** | -0.08 | 0.03 | -2.95 | .003 |
| **Unpredictability** | -0.01 | 0.02 | -0.56 | .58 |
| Health-information seeking online | 0.48 | 0.04 | 12.86 | < .001 |
| Self-rated health | 0.09 | 0.03 | 3.66 | < .001 |
| Sex (1 = male, 2 = female) | -0.03 | 0.02 | -1.50 | .13 |
| Age | -0.09 | 0.02 | -3.86 | < .001 |
| Education | -0.01 | 0.03 | -0.35 | .73 |
| Income | -0.08 | 0.03 | -2.87 | .004 |
| *Predicting IPC (reversed)* (mediator) |  |  |  |  |
| **Harshness** | 0.04 | 0.03 | 1.55 | .12 |
| **Unpredictability** | 0.09 | 0.03 | 3.33 | .001 |
| Health-information seeking online | -0.56 | 0.03 | -17.18 | < .001 |
| Self-rated health | -0.18 | 0.03 | -6.03 | < .001 |
| Sex (1 = male, 2 = female) | 0.02 | 0.02 | 0.79 | .43 |
| Age | -0.06 | 0.03 | -1.98 | .048 |
| Education | -0.00 | 0.03 | -0.09 | .93 |
| Income | -0.06 | 0.03 | -2.07 | .04 |
| *Predicting non-IPC (reversed)* (mediator) |  |  |  |  |
| **Harshness** | 0.08 | 0.03 | 3.07 | .002 |
| **Unpredictability** | 0.14 | 0.02 | 5.66 | < .001 |
| Health-information seeking online | -0.44 | 0.04 | -11.98 | < .001 |
| Self-rated health | -0.15 | 0.03 | -5.01 | < .001 |
| Sex (1 = male, 2 = female) | -0.02 | 0.03 | -0.81 | .42 |
| Age | -0.11 | 0.03 | -3.29 | .001 |
| Education | -0.03 | 0.03 | -0.80 | .42 |
| Income | -0.14 | 0.03 | -4.90 | < .001 |

*Note*: IPC = insight, planning, and control. Key predictor variables are in bold.
